# Manual of Nervous Diseases and an Introduction to Medical Electricity

**Published:** 1885-03

**Authors:** 


Manual of Nervous Diseases and an Introduction to Medical Electricity, by A. B. Arnold, M. D., Prof. of Diseases of the Nervous System and Clinical Medicine, College of Physicians and Surgeons, Baltimore, Md., with illustrations. New York: J. H. Vail & Co., 1885.

It is questionable whether greater temerity marks the undertaking to write a book connected with the nervous system or the attempt to criticise such a production. At the present stage of development in the knowledge of nervous diseases, he who essays to keep up with the advances in theory and practice cannot confine his attention to the old landmarks, but must find out the new lines of investigation and analyze closely the results of recent experimentation. The cerebral centres of peripheral local disturbances have recently been so precisely fixed as to actually lead to the extirpation of a glioma from the fissure of Rolando, based upon a diagnostic indication, and various other localizations of remote disturbances are well known. It has been ascertained by actual experiment that the films of nerves of entirely different origin and function, being respectively motor or sensory, when united, conveyed impulses peculiar to other motor or sensory nerves with which the connection was established. The vagus and hypoglossal, being of distinct origin and function, were divided by cutting the hypoglossal on one side close to its exit from the cranium in the dog, and the vagus at the thyroid gland. The peripheral end of the hypoglossal was sutured to the central end of the vagus by a silk thread through the neurilemma. The result proves that the motor fibres of the vagus united to similar fibre of the hypoglossal, and that the hypoglossal fibres conveyed impulses which were peculiar to the vagus apparatus. Irritation of the sinewy fibres in the hypoglossal trunk gave rise to impulses which were conveyed by the sensory fibres of the vagus to the vagus centres, thus forcing the interchange of function in nerves of different origin.

Whether our author is abreast of all that has been done in neurology, needs not to be here considered, as he claims only "to present in a concise manner the established facts and current theories relating to nervous affections." An interpolation on medical electricity in the body of this treatise might have been more appropriately introduced at the close among the curative agencies resorted to in the treatment of disorders of the nervous system. The failure to give any elucidation of the principles upon which electricity is made available for the correction of the derangements of the different nerve functions, should have been sufficient excuse for referring the reader to some concise explanation, such as that of Amidon, instead of marring the text with this chapter.

For the purposes of a text-book a methodic presentation of the fundamental elements of the cerebro-spinal and ganglionic nerves was requisite as a preliminary to understanding the various relations of their ramifications in the different organs, and we search in vain for this systematic outline of the nervous system.

In the details spread out upon many pages in the first half of the volume, the reader gathers up most of the information in regard to these divisions which might have been embodied in a single chapter. It was to have been expected that some classification should be adopted which was based upon the nature of the nerve influence propagated to different organs or to some special regions of the body, yet apart from the use of such general terms as neuralgia and neuritis, no such basis of distinction is recognized for the diseases of the nerve centres.

We have, however, in lieu of such a discrimination in the quality of the nerve agency at work in special disturbances, a division of nervous derangements based upon the designation of the parts involved, as the diseases of the spinal cord and its membranes, those of the brain and membranes, and what is styled classical neurosis.

In the long run, by a synthetic proceeding he includes what might have been more satisfactorily treated in the analytical form. That dynamic element of the nervous system which underlies all the vital forces of the organism has been overlooked entirely in this work, and as a consequence the modes of treatment adopted for the various modifications of nerve centres are for the most part empirical. The routine therapeutic measures are based largely upon the idea of relieving pain, and different preparations of opium occupy a conspicuous place among the proposed remedies. Instead of directing measures of relief to the cause of derangement they are addressed to the effects or consequences of the disease. Withal, much useful information is interlarded with the notices of various nervous affections; and though the treatise lacks that systematic classification which a manual should present, it may be read with profit by those interested in the details of treatment.

If the author should in another edition set aside the plan adopted in his annual course of lectures, and use the available materials collected in a scientific distribution based upon pathological and physiological principles, the value of the work would be enhanced.

